# Antitumor and Antibacterial Derivatives of Oridonin: A Main Composition of Dong-Ling-Cao

**DOI:** 10.3390/molecules21050575

**Published:** 2016-04-30

**Authors:** Dahong Li, Tong Han, Shengtao Xu, Tingting Zhou, Kangtao Tian, Xu Hu, Keguang Cheng, Zhanlin Li, Huiming Hua, Jinyi Xu

**Affiliations:** 1 Key Laboratory of Structure-Based Drug Design & Discovery, Ministry of Education, School of Traditional Chinese Materia Medica, Shenyang Pharmaceutical University, Shenyang 110016, China; lidahong0203@163.com (D.L.); hantong1221@163.com (T.H.); tingtingzhou1118@hotmail.com (T.Z.); kangtaotian@126.com (K.T.); huxu105@163.com (X.H.); lzl1030@hotmail.com (Z.L.); 2 State Key Laboratory of Natural Medicines and Department of Medicinal Chemistry, China Pharmaceutical University, Nanjing 210009, China; cpuxst@163.com; 3 State Key Laboratory of New-Tech for Chinese Medicine Pharmaceutical Processes, National Post-Doctoral Research Workstation, Jiangsu Kanion Pharmaceutical Co. Ltd., Lianyungang 222001, China; 4 State Key Laboratory for the Chemistry and Molecular Engineering of Medicinal Resources, School of Chemistry and Pharmacy, Guangxi Normal University, Guilin 541004, China

**Keywords:** *Isodon rubescens*, medicinal tea, diterpenoid, oridonin, medicinal chemistry

## Abstract

*Isodon rubescens* has been used as a traditional green tea for more than 1000 years and many medicinal functions of *I. rubescens* are also very useful, such as its well-known antitumor and antibacterial activities. Oridonin, a bioactive *ent*-kaurane diterpenoid, is the major ingredient of this medicinal tea. Herein, 22 novel oridonin derivatives were designed and synthesized. The antibacterial activity was evaluated for the first time. Compound **12** was the most promising one with MIC of 2.0 μg/mL against *B. subtilis*, which was nearly 3-fold stronger than positive control chloromycetin. The antiproliferative property was also assayed and compound **19** showed stronger activity than taxol. The apoptosis-inducing ability, cell cycle arrest effect at S phase and influence of mitochondrial membrane potential by **19** in CaEs-17 cancer cells were first disclosed. Based on the above results, the cell apoptosis induced by compound **19** in CaEs-17 cells was most probably involved in the intrinsic apoptotic pathway.

## 1. Introduction

**Figure 1:**
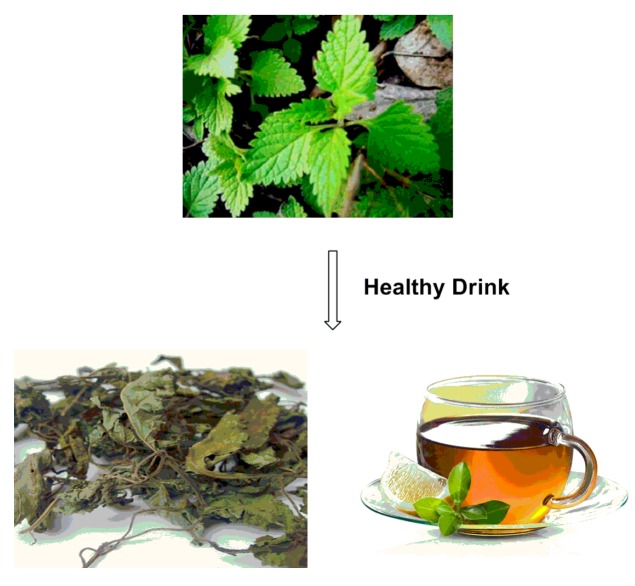
Tea drink of the leaves of *Isodon rubescens.*

*Isodon rubescens* (Chinese name Dong-ling-cao), belongs to the *Isodon* genus of the Labiatae family. It has been used as a tea drink for more than 1100 years from the Tang Dynasty (AD 618–907) in Taihang Mountain Area, and was recorded in a traditional Chinese book “Qi-xian-zhi” around the year 1660. The leaves are still used as a kind of tea (Figure 1) nowadays, especially in Henan Province of the People’s Republic of China. In the meantime, many medical functions of *I.* *rubescens* are very useful, such as antitumor and antibacterial activities. It is also a kind of antibiotic and antiphlogistic folk medicine and was first listed in Pharmacopoeia of the People’s Republic of China in the year 1977. The major chemical composition of *I. rubescens* is diterpenoids, such as oridonin, ponicidin, pedalitin, taibairubescensins A and B, xindongnins A and B, and so on [1,2,3,4,5,6,7,8]. Of these, oridonin is the major and most important ingredient of this medicinal tea. Numerous reports have shown that oridonin possesses remarkable antitumor activity both *in vitro* and *in vivo* [9,10,11]. Nevertheless, only a few studies have concerned the antibacterial (only antimycobacterial) activity of oridonin or its derivatives [12,13]. Our research group has carried out a lot of work into the components of *I. rubescens* or their derivatives, especially the antitumor activity of spirolactone-type 6,7-*seco*-kaurane diterpenoids, enmein-type 6,7-*seco*-kaurane diterpenoids and their derivatives [14,15,16,17]. Only a small number of studies could be found about the antibacterial and antitumor use of the major component oridonin or its derivatives in the field of medicinal chemistry.

For the above reasons, 22 oridonin derivatives were designed and synthesized. The antibacterial activity of oridonin and its derivatives against *Escherichia coli* (ATCC 25922), *Bacillus subtilis* (CMCC 63501), *Staphylococcus aureus* (ATCC 29213), and *Monilia albicans* (ATCC 10231) was evaluated for the first time. The antiproliferative properties were also assayed. The apoptosis-inducing ability, cell cycle arrest effect and influence of mitochondrial membrane potential by the typical derivative **19** in CaEs-17 cancer cells were further disclosed.

## 2. Results and Discussions

### 2.1. Synthesis of Compounds ***2**–**12*** and ***14**–**24***

**Scheme 1:**
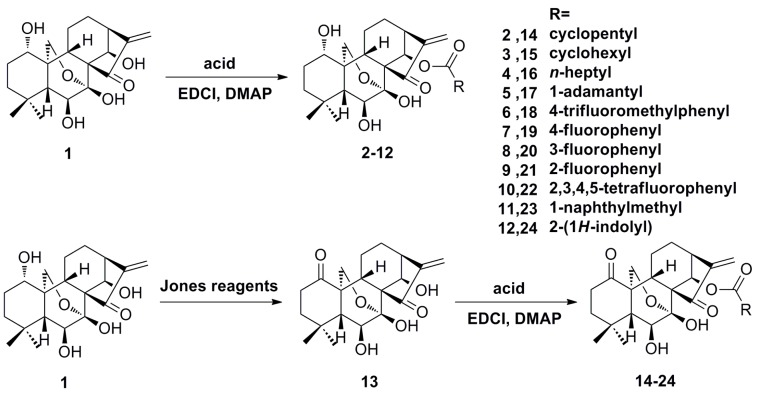
Synthetic routine of compounds **2**–**12** and **14**–**24**.

Compound **13** was synthesized from **1** by selected oxidation with Jones Reagent and no further purification was needed for the next step. Target compounds **2**–**12** and **14**–**24** were obtained by treatment of compound **1** or **13** with corresponding acid in the presence of EDCI and DMAP in dichloromethane (DCM) at room temperature for 8–12 h. 14-OH was first regio-selectively esterified in this reaction condition [14,18]. Flash chromatography could only be taken at the last step of the synthetic route of each target compound (Scheme 1).

### 2.2. Antimicrobial Activity

The antibacterial activity of **1**–**24** against *M. albicans*, *B. subtilis*, *S. aureus*, and *E. coli* were evaluated and listed in Table 1. Most of them were active against Gram-negative bacterium *S. aureus* and *B. subtilis*. No obvious inhibitory activity (MIC > 100 μg/mL) was observed in any of the synthetic derivatives against fungus *M. albicans* and Gram-negative bacterium *E. coli*. Oridonin (**1**) exhibited moderate potency against *S. aureus* and *B. subtilis* with MIC of 31.2 μg/mL. Among the derivatives **2**–**12**, **2** and **10** were weaker than parent compound **1**, and the others showed similar or better activity. As for **2**–**5** with R of aliphatic substituents, **4** was the strongest with R of *n*-heptyl and the MIC values were 15.6 and 3.9 μg/mL against *S. aureus* and *B. subtilis*, correspondingly. The fragments contained fluorine atoms were also introduced into diterpenoids scaffold (**6**–**10** and **18**–**22**), since fluorinated compounds always had unique biological and physicochemical properties, and they were widely spread in modern drugs. The derivatives with 4-trifluoromethylphenyl (**6**) and mono-fluorine substituted benzyl (**7**–**9**) exhibited similar potency and were stronger than that with multiple-fluorine substituted benzyl (**10**). **12** with R of 2-(1*H*-indolyl) was the most promising one with MICs of 3.9 and 2.0 μg/mL, respectively, which was similar as positive control chloromycetin against *S. aureus* and 3-fold stronger against *B. subtilis*. This confirmed that introducing hetero-*N* atom contained moistures into the natural lead molecules (usually only obtained C, H and O atoms) could always enhance the bioactivity and/or drug-like properties. 1-Position oxidized oridonin derivative (**13**) was weaker than oridonin, and the antibacterial activity of its derivatives (**14**–**24**) was also not as potent as the corresponding ones (**2**–**12**) of oridonin, while similar SARs could be concluded. According to the so called ‘rule of five’ proposed by Lipinski in 1997, Clog *p* values of less than 5 suggested appropriate membrane permeability for a potential drug candidate. All the designed derivatives showed Clog *p* values below 5 which could penetrate into cells to some extent and no further relationships between antimicrobial activity and Clog *p* values could be concluded.

### 2.3. Antiproliferative Activity

The antiproliferative activity of the derivatives together with their corresponding parent compounds **1** and **13** was evaluated against Bel-7402, K562, MGC-803 and CaEs-17 cells, and the results were listed in Table 2. All the synthetic derivatives showed better antiproliferative activity than their parent compounds **1** and **13**. 1-Position oxidized oridonin analogue (**13**) showed stronger antiproliferative activity than oridonin (**1**) against four selected cell lines with IC_50_ values of 2.98, 4.34, 3.98 and 7.23 μM. Most of the derivatives (**14**–**24**) of **13** with bigger Clog *p* values were also stronger than corresponding derivatives of **1**. Similar SARs could be concluded from the derivatives of **1** and **13**. Among **14**–**17** with R of aliphatic substituents, **17** with 1-adamantyl group showed bigger IC_50_ values against four tested cell lines than the other three with R of cyclopentyl, cyclohexyl or *n*-heptyl. Other pharmacophores like indolyl were used as well and **24** with R of 2-(1*H*-indolyl) showed the most potent cytotoxicity of all tested compounds against Bel-7402 cells with IC_50_ value of 0.81 μM. Therefore, in our further research, more derivatives with hetero-*N* atom will be designed and synthesized. Among the mono-fluorine substituted benzyl derivatives (**19**–**21**), **19** with 4-fluorophenyl substituent exhibited the strongest activity with IC_50_ values of 0.98, 0.29, 0.60, and 0.22 μM, correspondingly. The value against CaEs-17 cell line was the smallest one among all the derivatives. So, compound **19** was selected for further mechanism study against CaEs-17 cells.

### 2.4. Apoptosis-Inducing Ability of ***19*** in CaEs-17 Cell Line

**Figure 2:**
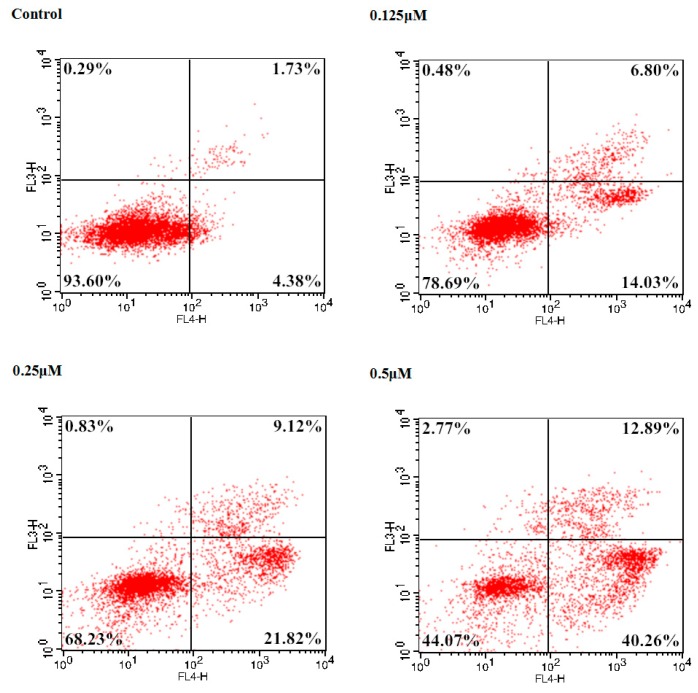
Apoptosis-inducing ability of compound **19** in CaEs-17 cell line.

Apoptosis is a process of programmed cell death, and cancer cells usually have an abnormal ability of proliferation, mainly due to the defective apoptosis. Thus, activation of apoptosis could reduce the accumulation of cancer cells. In order to examine the involvement, at least in part, of apoptosis in the loss of cancer cell viability of compound **19** in CaEs-17 cells, an annexin V-FITC/PI binding assay was carried out. CaEs-17 cells were treated with different concentrations of **19** and percentages of apoptotic cells were determined by flow cytometry. As shown in (Figure 2), **19** exhibited potent dose-dependent activity in the induction of apoptosis. Treatment of CaEs-17 cells with **19** at 0.125, 0.25, and 0.5 μM, apoptotic cell rates (early and late) were 20.83, 30.94, and 53.15%, as compared with 6.11% in an untreated vehicle control, indicating that **19** was able to induce apoptotic cell death in CaEs-17 cells.

### 2.5. Cell Cycle Effect on CaEs-17 Cells by ***19***

**Figure 3:**
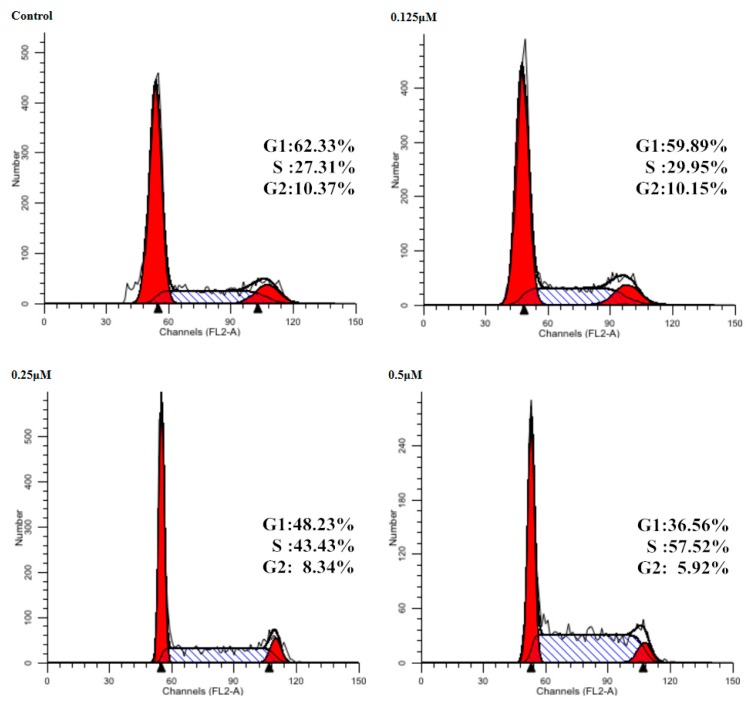
Cell cycle effect on CaEs-17 cells of compound **19**.

The cell cycle is the series of events that take place in a cell, leading to its division and duplication (replication). The apoptosis inducing activity of compound **19** was also characterized by flow cytometric analysis of the DNA profile. CaEs-17 cells were treated with compound **19** at concentrations of 0.125, 0.25, and 0.5 μM, which resulted in accumulation of 29.95%, 43.43%, and 57.52% of cells at the S phase, respectively, compared with the untreated cells of 27.31% (Figure 3). There were also a decline of G_1_ phase cells of 59.89%, 48.23%, and 36.56%, and G_2_ phase cells of 10.15%, 8.34% and 5.92%, respectively. So, compound **19** influenced cell-cycle progression of CaEs-17 cells at low sub-micromolar concentrations and arrested at S phase.

### 2.6. Effect on Mitochondrial Membrane Potential

**Figure 4:**
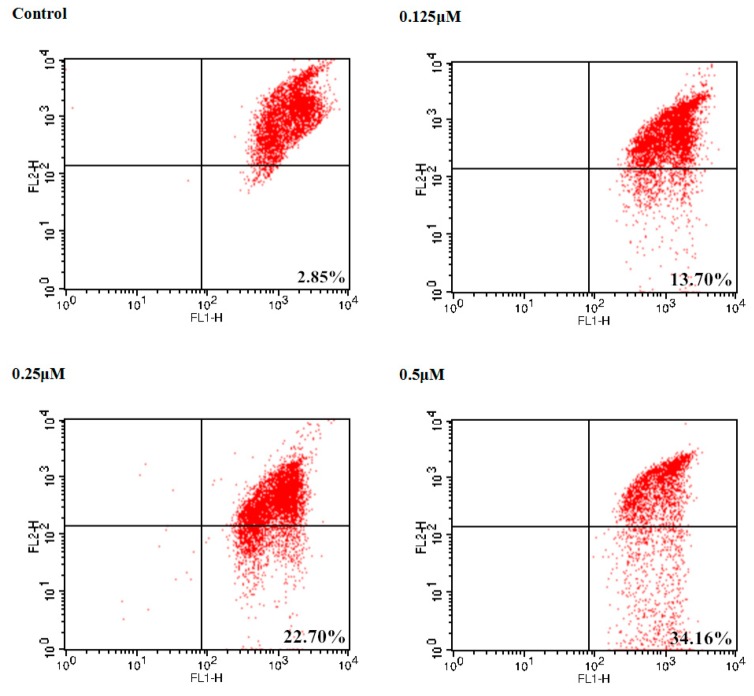
Effect on mitochondrial membrane potential of CaEs-17 cells by compound **19**.

In order to determine the involvement of mitochondrial in compound **19** mediated apoptosis in CaEs-17 cells, the changes on mitochondrial membrane potential were monitored by flow cytometry. CaEs-17 cells were incubated with different concentrations (0, 0.125, 0.25, and 0.5 μM) of compound **19** and the percentage of apoptotic cells increased in a concentration-dependent fashion (2.85%, 13.70%, 22.70% and 34.16%, respectively, Figure 4). These results indicated that the treatment of compound **19** in CaEs-17 cells triggered the collapse of mitochondrial membrane potentials and induced apoptosis.

## 3. Materials and Methods

### 3.1. Chemistry

#### 3.1.1. General

All the solvents and chemicals were purchased from Yuwang Chemical Industries, Ltd. (Shenyang, China) and not further purified. 3-(4,5-Dimethylthiazol-2-yl)-2,5-diphenyltetrazolium bromide (MTT), and fetal bovine serum (FBS) were bought from Sigma Aldrich (Sigma Chemical Co., Shanghai, China). Column chromatography was performed with silica gel (200–300 mesh, Qingdao Ocean Chemical Co. Ltd., Qingdao, China). TLC plates were precoated with silica gel GF 254 (Qingdao Ocean Chemical Co. Ltd.). Melting points (m.p.) were measured with XT-4 micro melting point apparatus and uncorrected. NMR was recorded with a Bruker AV-300 or AV-500 spectrometer (Bruker Corp., Karlsruhe, Germany) using TMS as an internal standard. Mass spectra were obtained using FTMS-2000 (Thermo Fisher Scientific, Waltham, MA, USA).

#### 3.1.2. General Procedure to Synthesize **2**–**12** and **14**–**24**

Compound **13** (72 mg, 0.2 mmol) was synthesized from **1** by oxidation with Jones Reagent and no further purification was needed for the next step [14]. Compound **1** or **13** (72 mg, 0.2 mmol) was dissolved in 10 mL of dichloromethane and mixed with corresponding acid (0.24 mmol), EDCI and DMAP. The reaction was stirred at room temperature and monitored by TLC. After 6–8 h, the mixture was poured into 10 mL of 10% HCl, and then extracted with DCM three times (each 10 mL). The organic layer was combined, washed with water and saturated brine, sequentially, then dried over anhydrous Na_2_SO_4_, and concentrated *in vacuo*. The crude product was purified by column chromatography (MeOH/DCM 1:150 *v*/*v*) to give the target compounds **2**–**12** and **14**–**24**.

*Compound* **19**: white solid, 24% yield, m.p. 114–116 °C; IR (KBr) υ_max_ 3404, 2957, 2361, 1707, 1643, 1604, 1508, 1458, 1280, 1242, 1155, 1080, 1051, 965, 909 cm^−1^; ^13^C-NMR (DMSO-*d*_6_, 125 MHz), *δ* (ppm) 211.47, 205.02, 166.92, 164.25, 149.59, 132.39, 132.31, 126.06, 122.07, 115.67, 115.50, 96.91, 74.91, 73.46, 64.93, 61.50, 59.87, 50.78, 48.59, 41.63, 38.41, 35.73, 32.84, 30.49, 30.06, 23.14, 19.20; ^1^H-NMR (CDCl_3_, 500 MHz), δ (ppm) 7.95 (2H, m, Ar-H), 7.06 (2H, d, *J* = 8.7 Hz, Ar-H), 6.30 (1H, s, 17-CH_2_), 6.10 (1H, s, 14-CH), 5.64 (1H, s, 17-CH_2_), 5.48 (1H, d, *J* = 11.5 Hz, 6-OH), 4.34, 4.03 (each 1H, d, *J*_A_ = *J*_B_ = 9.5 Hz, 20-CH_2_), 4.12 (1H, s, 7-OH), 3.77 (1H, m, 6-CH), 3.25 (1H, d, *J* = 9.9 Hz, 13-CH); MS (ESI) *m/z*: 485.2 [M + H]^+^, 507.2 [M + Na]^+^, 523.2 [M + K]^+^.

### 3.2. Biology

#### 3.2.1. Antibacterial Assay

The antibacterial activity against *M. albicans*, *S. aureus*, *B. subtilis*, and *E. coli* were evaluated. Generally, the minimal inhibitory concentrations (MICs) were measured by micro-broth dilution method. The stock solution of target compounds was diluted and added into microtitration plates. The specified concentration of fungus or bacterium was added and incubated. The MIC value was defined as the lowest concentration of compound at which no growth was observed [19,20].

#### 3.2.2. MTT Assay

Bel-7402 (human hepatoma), K562 (human leukemia), MGC-803 (human esophageal cancer), and CaEs-17 (human gastric cancer) cell lines were obtained from the American Type Culture Collection (Manassas, VA, USA). The cell lines were cultured in RPMI 1640 or DMEM with high glucose or low glucose supplemented with 10% FBS at 37 °C in 5% CO_2_. The antiproliferative activity was performed by the MTT method [21,22]. In brief, Bel-7402 cells were placed in 96-well plates and incubated for 24 h. Concentrations of the compounds (purity > 98%) were added and incubated for another 96 h. After that, MTT solution was added, and the absorbance at 570 nm was measured. Taxol was used as the positive control. The results were obtained from three independent experiments carried out in duplicate. All measurements of antiproliferative activities were repeated in triplicate and data were expressed as mean ± SD (standard deviations). An ANOVA test using SPSS 16.0 (Statistical Program for Social Sciences, SPSS Inc., Chicago, IL, USA) was used to analyze the experimental data.

#### 3.2.3. Cell Apoptosis

The CaEs-17 cells were incubated with compound **19** at certain concentrations for 3 days and apoptosis was analyzed using Annexin-V and propidium iodide (PI) double staining method by flow cytometry similar as previous reports [23] according to operation manual.

#### 3.2.4. Effect of Cell Cycle

CaEs-17 cells were plated in 6-well plates and incubated for 24 h, and then certain concentrations of compound **19** were added. After 48 h treatment, cells were fixed with 70% ethanol, treated with RNase, and stained with PI. DNA content was measured using a flow cytometer [24].

#### 3.2.5. Mitochondrial Membrane Potential

CaEs-17 cells were cultured overnight and incubated with the test compound or vehicle in triplicate for 48 h. The cells were stained with the lipophilic cationic dye (JC-1), according to the manufacturer’s instruction (Keygen, KGA601, Nanjing, China). The percentage of cells with healthy or collapsed mitochondrial membrane potentials was monitored by flow cytometry [25].

## 4. Conclusions

In this study, 22 derivatives (**2**–**12** and **14**–**24**) of oridonin (**1**) and 1-position oxidized oridonin (**13**) were designed and synthesized. All the title compounds were first evaluated for their antimicrobial activity against *M. albicans*, *B. subtilis*, *S. aureus* and *E. coli*, and antiproliferative activity against Bel-7402, K562, MGC-803 and CaEs-17 cells. Preliminary SARs were also determined. Compound **12** with R of 2-(1*H*-indolyl) was the most promising antimicrobial candidate with MICs of 3.9 and 2.0 μg/mL against *S. aureus* and *B. subtilis*, respectively. Derivative **19** with R of 4-fluorophenyl showed the smallest IC_50_ value of 0.22 μM among all the derivatives against CaEs-17 cell line, which was almost 50-fold stronger than lead oridonin (**1**) and nearly 1-fold more potent than taxol (0.43 μM). The apoptosis-inducing effect of **19** was further investigated using CaEs-17 cells. It was found that **19** arrested CaEs-17 cell cycle at S phase and incubation with **19** induced apoptosis and mitochondrial depolarization at low sub-micromolar concentrations in dose-dependent manner. Apoptosis was divided into intrinsic and extrinsic pathways. The intrinsic pathway mainly depended on the loss of mitochondria membrane potential. Therefore, the cell apoptosis induced by **19** in CaEs-17 cells was most probably involved in the intrinsic apoptotic pathway. It was expected that the remarkable biological profile of oridonin analogs made them possible as promising candidates for the development of novel agents from functional foods.

## Figures and Tables

**Table 1:** The antimicrobial activity of oridonin and its derivatives (MIC μg/mL).

Compound	*E. coli*	*S. aureus*	*B. subtilis*	*M. albicans*	Clog *p* ^2^
**1**	>100	31.2	31.2	>100	−1.70
**2**	>100	62.5	31.2	>100	0.65
**3**	>100	31.2	15.6	>100	1.21
**4**	>100	15.6	3.9	>100	2.35
**5**	>100	15.6	7.8	>100	1.84
**6**	>100	31.2	15.6	>100	1.65
**7**	>100	31.2	15.6	>100	0.91
**8**	>100	31.2	15.6	>100	0.91
**9**	>100	15.6	15.6	>100	0.47
**10**	>100	62.5	31.2	>100	0.76
**11**	>100	15.6	7.8	>100	1.96
**12**	>100	3.9	2.0	>100	1.17
**13**	>100	62.5	31.2	>100	−0.06
**14**	>100	>100	>100	>100	1.70
**15**	>100	62.5	31.2	>100	2.26
**16**	>100	15.6	7.8	>100	3.40
**17**	>100	15.6	15.6	>100	2.89
**18**	>100	62.5	15.6	>100	2.70
**19**	>100	31.2	15.6	>100	1.96
**20**	>100	31.2	15.6	>100	1.96
**21**	>100	31.2	15.6	>100	1.52
**22**	>100	>100	62.5	>100	1.81
**23**	>100	31.2	7.8	>100	3.01
**24**	>100	3.9	3.9	>100	2.22
chloromycetin	3.9	3.9	7.8	NT ^1^	NT
fluconazole	NT	NT	NT	3.91	NT

^1^ NT, not test; ^2^ Clog *p* values were calculated by ChemBioDraw Ultra 12.0 (ChemBridge Corp., San Diego, CA, USA).

**Table 2:** Antiproliferative activity of compounds **1**–**24** against Bel-7402, K562, MGC-803 and CaEs-17 human cancer cells (IC_50_ μM; mean ± S.D.).

Compound	Bel-7402	K562	MGC-803	CaEs-17
**1**	7.48 ± 0.53	4.76 ± 0.32	5.69 ± 0.39	11.03 ± 1.02
**2**	1.18 ± 0.03	2.03 ± 0.17	1.18 ± 0.21	3.36 ± 0.22
**3**	1.01 ± 0.04	1.97 ± 0.18	1.12 ± 0.10	3.25 ± 0.31
**4**	0.96 ± 0.06	1.83 ± 0.23	1.08 ± 0.06	3.20 ± 0.29
**5**	0.97 ± 0.11	1.84 ± 0.36	1.14 ± 0.23	3.16 ± 0.37
**6**	1.63 ± 0.81	0.25 ± 0.02	0.81 ± 0.10	0.61 ± 0.13
**7**	1.07 ± 0.52	0.31 ± 0.04	0.37 ± 0.04	0.43 ± 0.10
**8**	1.13 ± 0.14	0.37 ± 0.07	0.61 ± 0.01	0.28 ± 0.22
**9**	1.39 ± 0.72	0.59 ± 0.19	1.03 ± 0.55	0.29 ± 0.04
**10**	3.80 ± 0.92	2.66 ± 0.13	4.02 ± 0.75	7.23 ± 1.03
**11**	0.90 ± 0.02	1.87 ± 0.07	1.37 ± 0.09	3.92 ± 0.36
**12**	0.82 ± 0.22	1.74 ± 0.23	1.12 ± 0.17	3.63 ± 0.29
**13**	2.98 ± 0.14	4.34 ± 0.04	3.98 ± 0.66	7.23 ± 0.73
**14**	0.98 ± 0.06	1.77 ± 0.13	1.31 ± 0.14	2.72 ± 0.30
**15**	0.93 ± 0.11	1.76 ± 0.24	1.08 ± 0.09	2.56 ± 0.25
**16**	0.95 ± 0.07	1.81 ± 0.24	1.09 ± 0.13	3.13 ± 0.22
**17**	0.99 ± 0.10	1.91 ± 0.45	1.17 ± 0.15	3.46 ± 0.44
**18**	2.81 ± 0.41	0.86 ± 0.03	1.02 ± 0.38	0.89 ± 0.30
**19**	0.98 ± 0.05	0.29 ± 0.05	0.60 ± 0.40	0.22 ± 0.09
**20**	1.66 ± 0.39	0.35 ± 0.06	0.87 ± 0.05	0.64 ± 0.11
**21**	1.45 ± 0.42	0.47 ± 0.01	0.90 ± 0.22	0.51 ± 0.03
**22**	3.17 ± 0.65	2.16 ± 0.37	3.94 ± 0.71	8.55 ± 0.80
**23**	1.07 ± 0.13	1.72 ± 0.24	1.25 ± 0.16	3.62 ± 0.18
**24**	0.81 ± 0.08	1.66 ± 0.26	1.09 ± 0.24	3.57 ± 0.16
**Taxol ^1^**	1.89 ± 0.09	0.41 ± 0.02	0.85 ± 0.06	0.43 ± 0.03

^1^ Taxol was used a positive control. Data were means ± SD of three experiments.

## Supplementary Materials

Supplementary materials of NMR spectra of **1**, **13** and **19**, and spectra data of other target compounds could be found online at: http://www.mdpi.com/1420-3049/21/5/575/s1.

## Abbreviations

The following abbreviations are used in this manuscript:

SAR: Structure Activity Relationship
^1^H-NMR: Proton Nuclear Magnetic Resonance
TMS: Tetramethlysilane
MS: Mass Spectrometry
ESI: Electrospray Ionization
DCM: Dichloromethane
MTT: 3-(4,5-Dimethylthiazol-2-yl)-2,5-diphenyltetrazolium bromide
FBS: Fetal Bovine Serum
MIC: Minimal Inhibitory Concentration
NT: Not Test
