# Supplementary material for: Antitumor and Antibacterial Derivatives of Oridonin: A Main Composition of Dong-Ling-Cao
Source: Molecules. 2016 Apr 30;21(5):575. doi: 10.3390/molecules21050575 (PMC6272837; doi:10.3390/molecules21050575)
Supplement: Supplementary file 1 [file molecules-21-00575-s001.pdf]

Supplementary Materials: Antitumor and Antibacterial Derivatives of Oridonin: A Main Composition of Dong-Ling-Cao

Dahong Li, Tong Han, Shengtao Xu, Tingting Zhou, Kangtao Tian, Xu Hu, Keguang Cheng, Zhanlin Li, Huiming Hua and Jinyi Xu

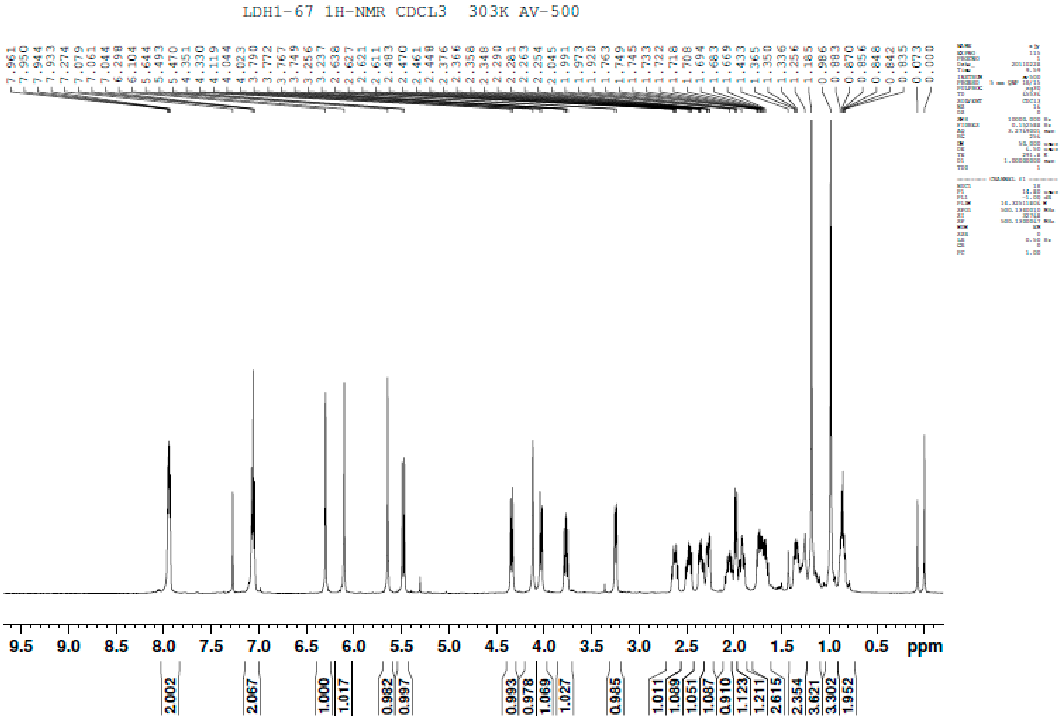

Figure S1. ^{1}H-NMR spectrum of compound **19** (500 MHz, $CDCl_3$).

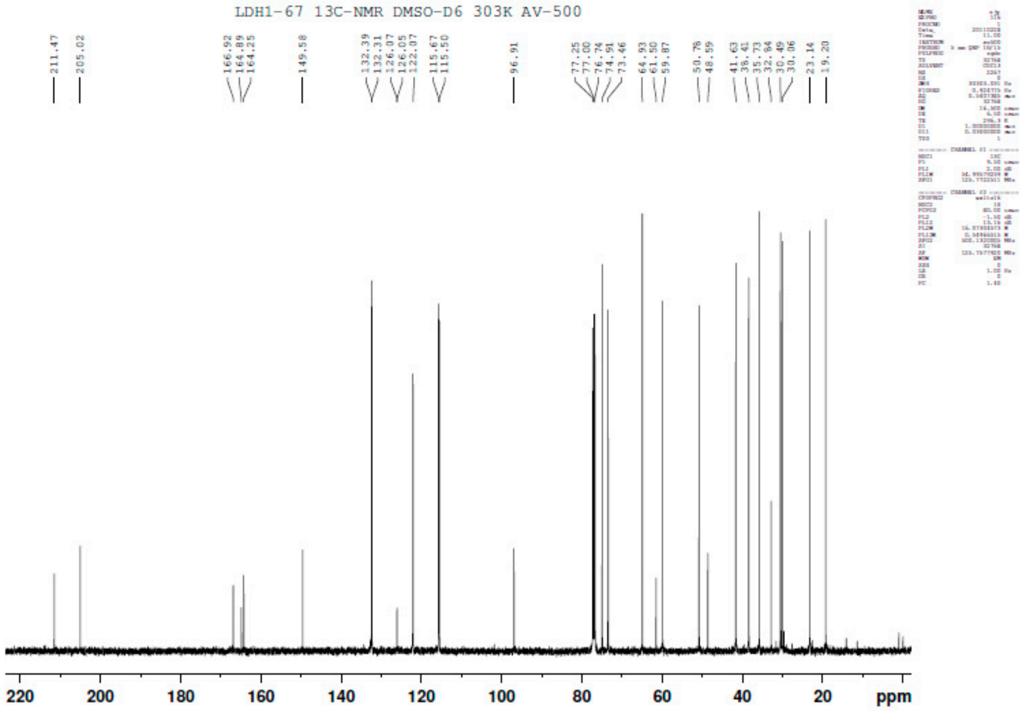

Figure S2. ^{13}C-NMR spectrum of compound **19** (125 MHz, DMSO-*d*$_6$).

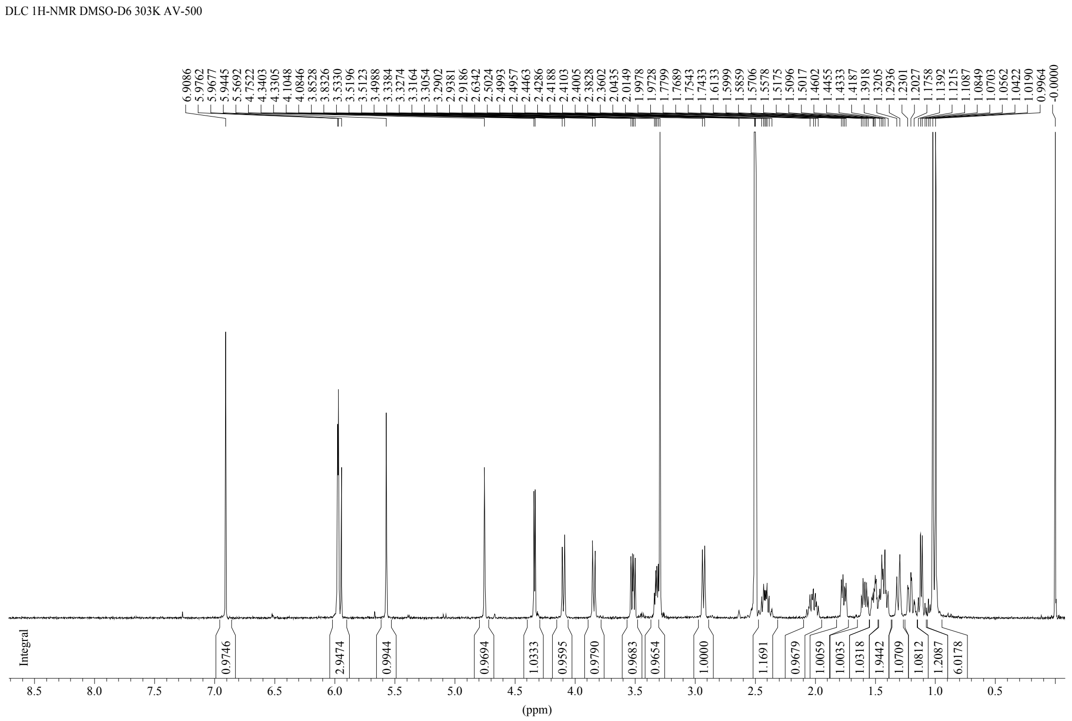

Figure S3. ^{1}H-NMR spectrum of oridonin (500 MHz, DMSO-*d*$_6$).

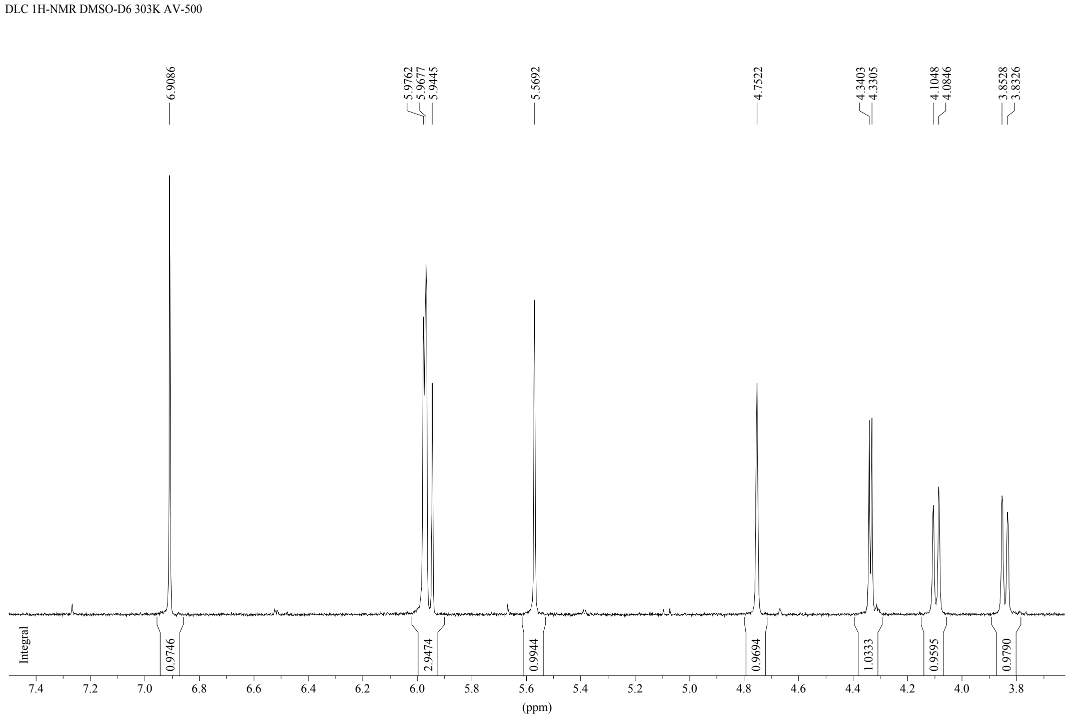

Figure S4. Region amplified ^{1}H-NMR spectrum 2-1 of oridonin (500 MHz, DMSO-d_6).

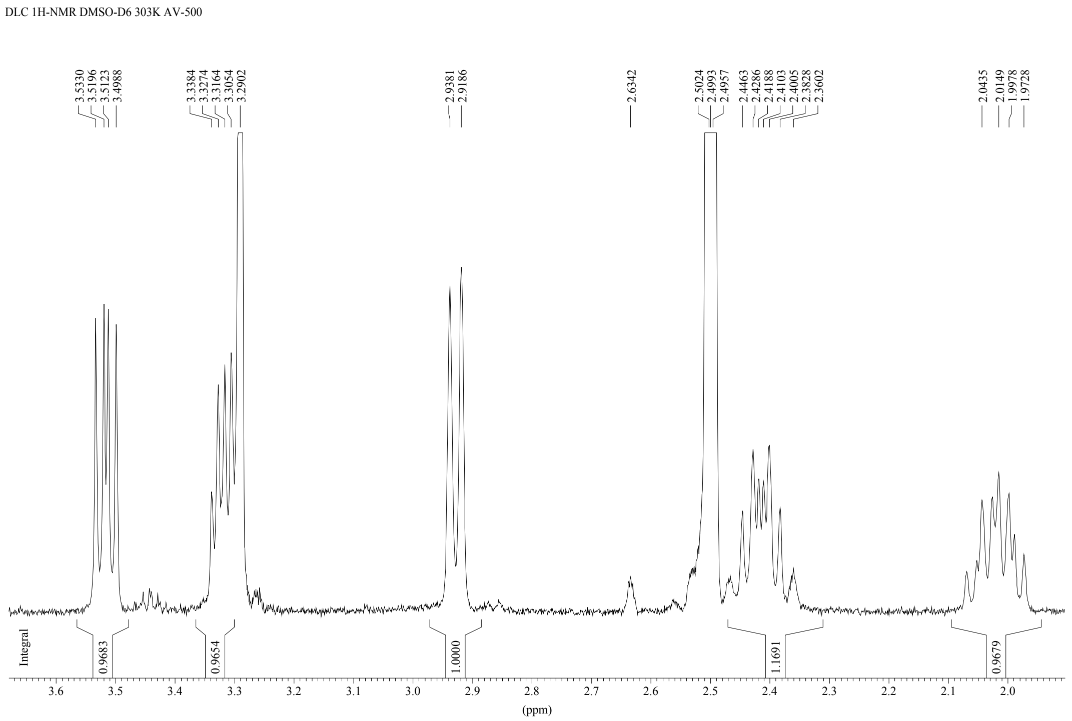

Figure S5. Region amplified ^{1}H-NMR spectrum 2-2 of oridonin (500 MHz, DMSO-d_6).

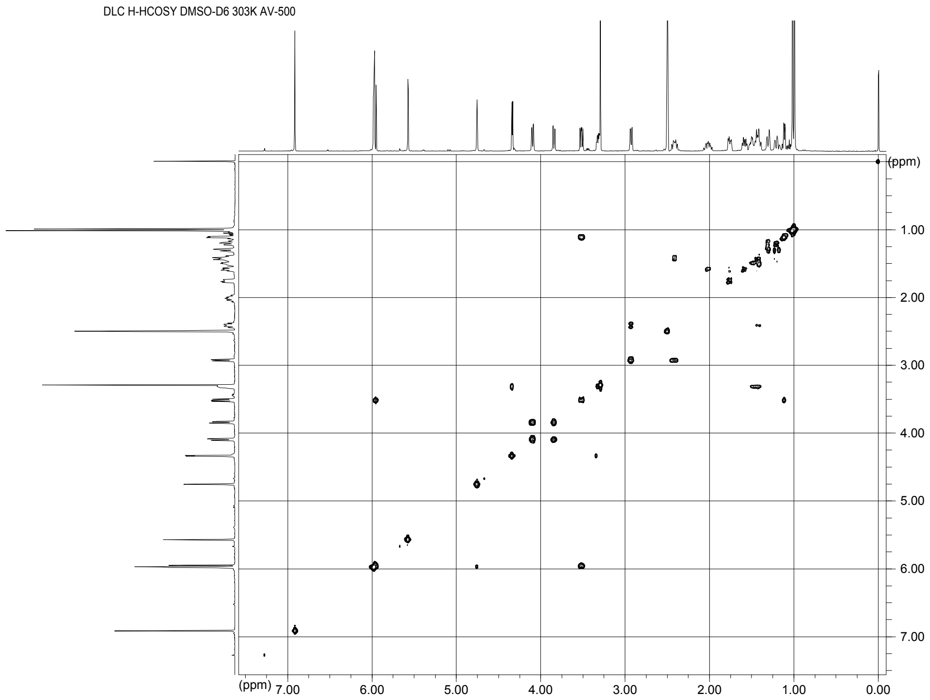

Figure S6. H-H COSY spectrum of oridonin (500 MHz, DMSO-d_6).

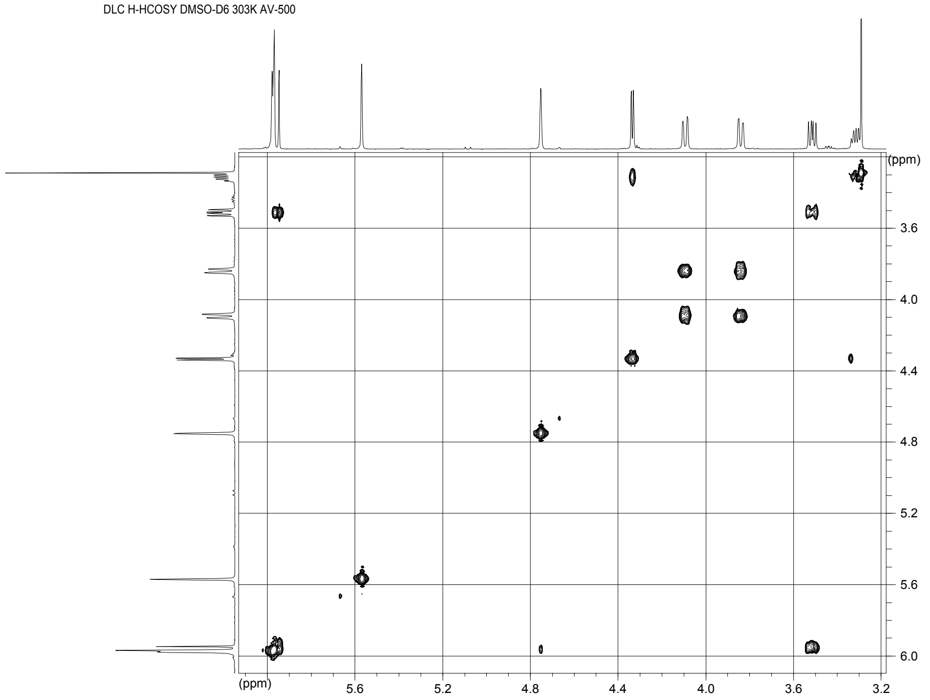

Figure S7. Region amplified H-H COSY spectrum 5-1 of oridonin (500 MHz, DMSO-d_6).

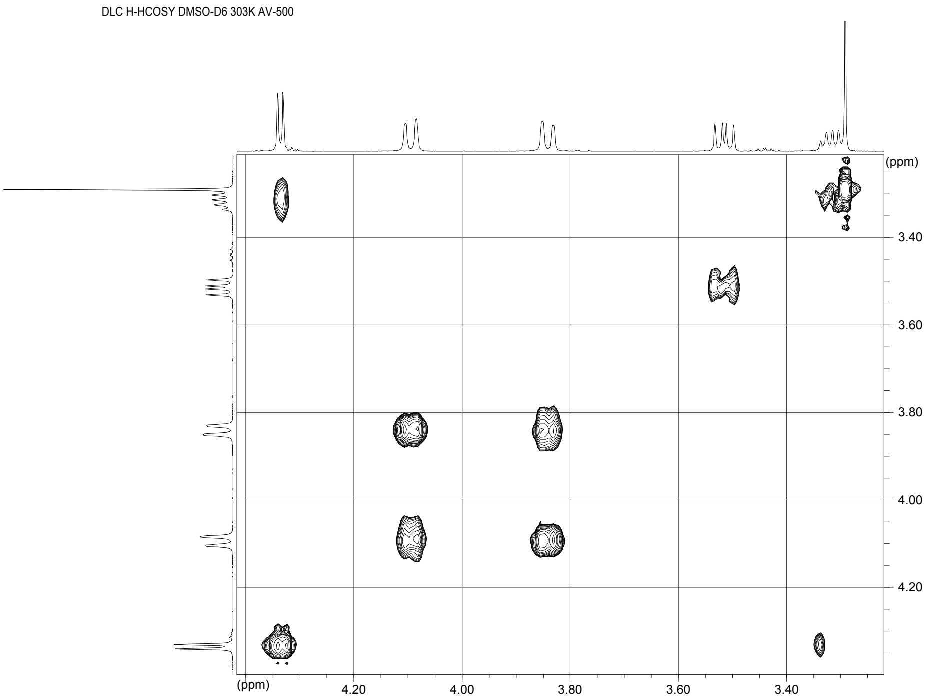

Figure S8. Region amplified H-H COSY spectrum 5-2 of oridonin (500 MHz, DMSO-d_6).

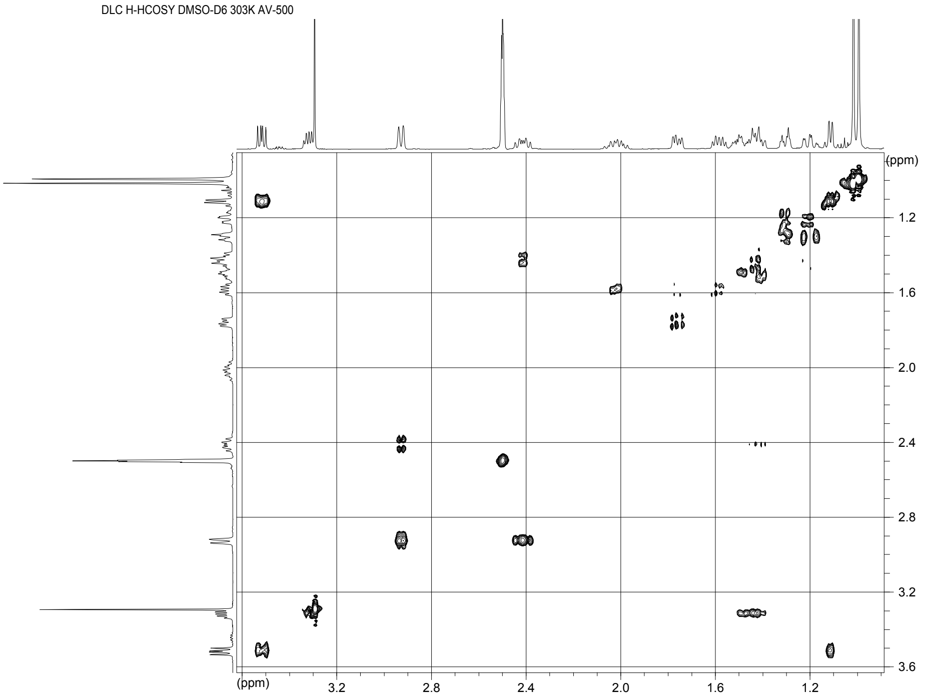

Figure S9. Region amplified H-H COSY spectrum 5-3 of oridonin (500 MHz, DMSO-d_6).

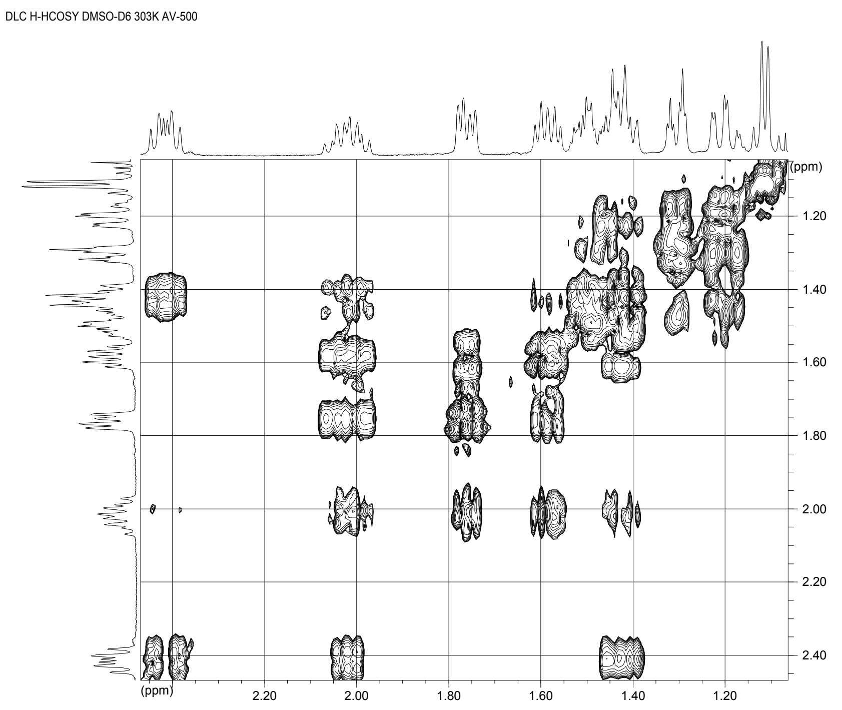

Figure S10. Region amplified H-H COSY spectrum 5-4 of oridonin (500 MHz, DMSO-d_6).

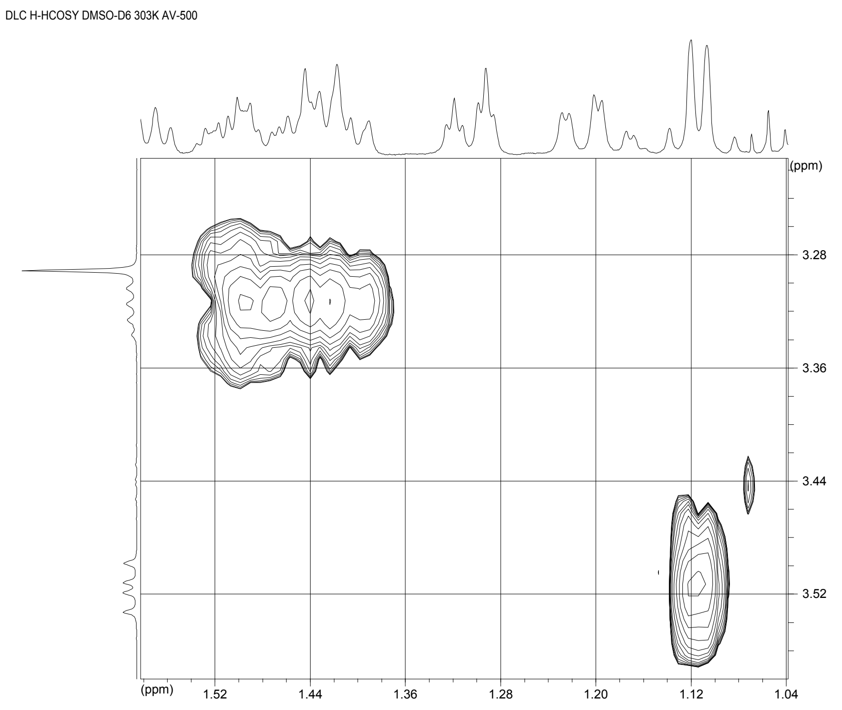

Figure S11. Region amplified H-H COSY spectrum 5-5 of oridonin (500 MHz, DMSO-d_6).

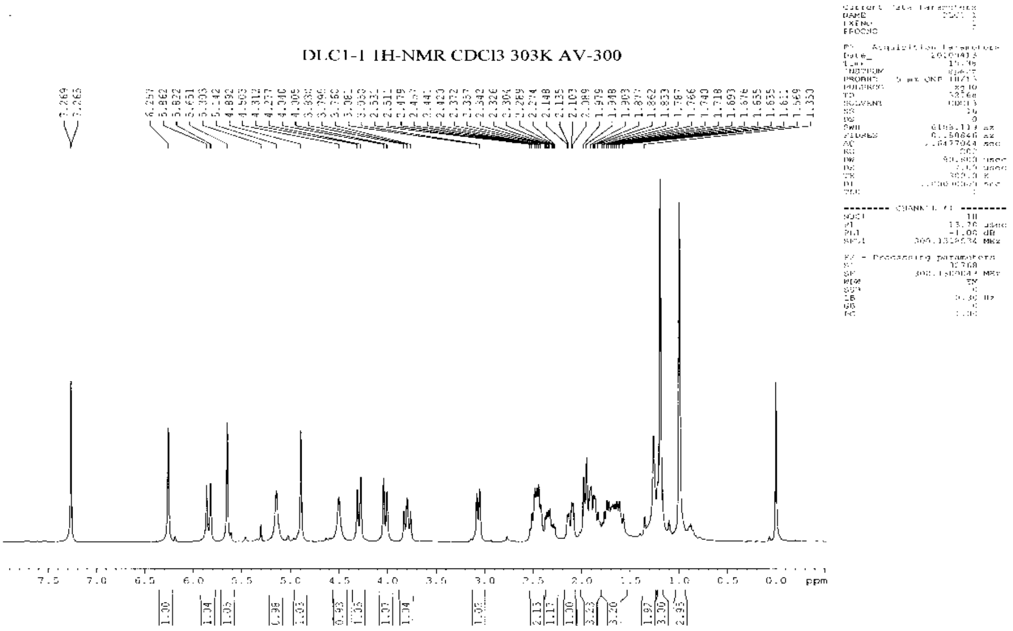

Figure S12. ^{1}H-NMR spectrum of **13** (300 MHz, $CDCl_3$).

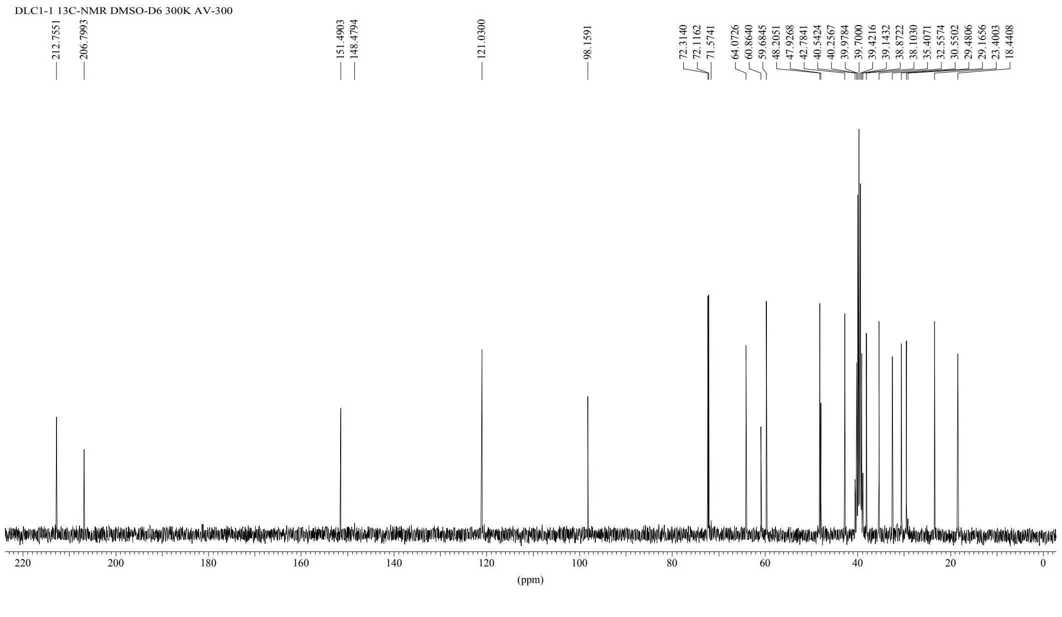

Figure S13. ^{13}C-NMR spectrum of **13** (75 MHz, DMSO-d_6).

Spectrum data of compounds **2–12**, **14–18** and **20–24**:

Compound **2**: yellow solid, 37 % yield, m.p. 99-101 °C; IR (KBr) υ_{max} 3397, 2953, 2919, 2870, 2850, 1712, 1458, 1174, 1061, 946 cm^{-1}; ^{13}C-NMR ($CDCl_3$, 100 MHz), δ (ppm) 206.60, 174.87, 149.86, 119.91, 96.35, 77.24, 73.66, 73.46, 63.41, 61.70, 59.98, 54.49, 43.81, 41.30, 41.09, 38.64, 33.78, 32.52, 30.37, 30.09, 29.84, 29.70, 29.60, 25.75, 21.62, 19.71; ^{1}H-NMR ($CDCl_3$, 300 MHz), δ (ppm) 6.14 (1H, s, 17-CH_2), 6.09 (1H, d, J = 12.5 Hz, 6-OH), 5.79 (1H, s, 14-CH), 5.48 (1H, s, 17-CH_2), 4.44 (1H, s, 1-OH), 4.31, 4.08 (each 1H, d, J_A = J_B = 9.5 Hz, 20-CH_2), 3.80 (1H, m, 6-CH), 3.50 (1H, m, 1-CH), 3.19 (1H, d, J = 16.5 Hz, 13-CH); MS (ESI) m/z: 461.3 [M + H]$^+$, 483.3 [M + Na]$^+$.

Compound **3**: yellow oil, 34 % yield; IR (KBr) υ_{max} 3424, 2920, 2851, 1730, 1711, 1645, 1451, 1384, 1245, 1170, 938, 783 cm^{-1}; ^{13}C-NMR ($CDCl_3$, 100 MHz), δ (ppm) 206.55, 174.25, 149.84, 119.91, 96.34, 77.23,

76.34, 73.68, 73.49, 63.39, 61.69, 59.97, 54.50, 43.31, 41.31, 41.12, 38.64, 33.78, 32.53, 30.37, 30.10, 29.37, 28.90, 25.48, 25.24, 25.11, 21.62, 19.73; ^{1}H-NMR ($CDCl_3$, 300 MHz), δ (ppm) 6.15 (1H, s, 17-CH_2), 6.10 (1H, d, J = 12.5 Hz, 6-OH), 5.80 (1H, s, 14-CH), 5.48 (1H, s, 17-CH_2), 4.32 (1H, s, 1-OH), 4.31, 4.10 (each 1H, d, J_A = J_B = 9.5 Hz, 20-CH_2), 3.81 (1H, m, 6-CH), 3.52 (1H, m, 1-CH), 3.19 (1H, d, J = 16.5 Hz, 13-CH); MS (ESI) *m/z*: 475.3 $[M + H]^+$, 497.3 $[M + Na]^+$.

Compound **4**: yellow oil, 38 % yield; IR (KBr) υ_{max} 3379, 2926, 2854, 1712, 1644, 1459, 1270, 1168, 1061, 943, 911, 812 cm^{-1}; ^{13}C-NMR ($CDCl_3$, 100 MHz), δ (ppm) 206.61, 172.11, 149.83, 120.02, 96.26, 77.25, 76.89, 73.77, 73.41, 63.42, 61.75, 59.88, 54.51, 41.30, 41.17, 38.63, 34.59, 33.76, 32.52, 31.59, 30.44, 30.04, 28.92, 28.80, 24.66, 22.55, 21.60, 19.71, 14.05; ^{1}H-NMR ($CDCl_3$, 300 MHz), δ (ppm) 6.17 (1H, s, 17-CH_2), 6.14 (1H, s, 6-OH), 5.81 (1H, s, 14-CH), 5.48 (1H, s, 17-CH_2), 4.35 (1H, s, 1-OH), 4.30, 4.08 (each 1H, d, J_A = J_B = 10.2 Hz, 20-CH_2), 3.76 (1H, m, 6-CH), 3.50 (1H, m, 1-CH), 3.19 (1H, d, J = 9.3 Hz, 13-CH); MS (ESI) *m/z*: 491.3 $[M + H]^+$.

Compound **5**: white solid, 22 % yield, m.p. 180-182 °C; IR (KBr) υ_{max} 3448, 2918, 2849, 1724, 1709, 1642, 1411, 1080, 1053, 979, 968, 730 cm^{-1}; ^{13}C-NMR ($CDCl_3$, 100 MHz), δ (ppm) 206.55, 175.90, 149.89, 119.70, 96.39, 77.23, 73.60, 73.49, 63.40, 61.66, 60.10, 54.53, 41.32, 41.05, 40.84, 38.63, 36.15, 33.80, 32.53, 31.93, 30.29, 30.13, 29.71, 29.37, 27.64, 22.70, 21.60, 19.72; ^{1}H-NMR ($CDCl_3$, 300 MHz), δ (ppm) 6.14 (1H, s, 17-CH_2), 6.08 (1H, d, J = 10.5 Hz, 6-OH), 5.76 (1H, s, 14-CH), 5.46 (1H, s, 17-CH_2), 4.29, 4.08 (each 1H, d, J_A = J_B = 10.5 Hz, 20-CH_2), 4.09 (1H, s, 1-OH), 3.81 (1H, m, 6-CH), 3.76 (1H, m, 1-CH), 3.16 (1H, d, J = 10.2 Hz, 13-CH); MS (ESI) *m/z*: 527.3 $[M + H]^+$, 549.3 $[M + Na]^+$.

Compound **6**: white solid, 22 % yield, m.p. 180-182 °C; IR (KBr) υ_{max} 3448, 2918, 2849, 1724, 1709, 1642, 1411, 1080, 1053, 979, 968, 730 cm^{-1}; ^{13}C-NMR ($CDCl_3$, 100 MHz), δ (ppm) 206.53, 164.29, 150.00, 133.13, 130.20, 125.48, 125.44, 120.43, 96.02, 77.22, 76.28, 74.78, 73.56, 63.45, 62.23, 59.37, 54.84, 41.60, 41.40, 38.66, 33.73, 32.66, 30.58, 30.06, 21.78, 20.04; ^{1}H-NMR ($CDCl_3$, 300 MHz), δ (ppm) 8.05 (2H, d, J = 8.2 Hz, Ar-H), 7.65 (2H, d, J = 8.2 Hz, Ar-H), 6.21 (1H, s, 17-CH_2), 6.17 (1H, s, 14-CH), 6.07 (1H, d, 6-OH), 5.54 (1H, s, 17-CH_2), 4.38, 4.09 (each 1H, dd, J_A = J_B = 10.2 Hz, 20-CH_2), 3.78 (1H, s, 1-OH), 3.76 (1H, m, 6-CH), 3.54 (1H, m, 1-CH), 3.27 (1H, d, J = 9.6 Hz, 13-CH), 1.13 (3H, s, 18-CH_3), 1.11 (3H, s, 19-CH_3); MS (ESI) *m/z*: 527.3 $[M + H]^+$, 549.3 $[M + Na]^+$.

Compound **7**: white solid, 59 % yield, m.p. 237-239 °C; IR (KBr) υ_{max} 3450, 3349, 2966, 2933, 1711, 1643, 1604, 1508, 1280, 1115, 1092, 1060, 968, 856, 767 cm^{-1}; ^{13}C-NMR ($CDCl_3$, 100 MHz), δ (ppm) 206.67, 164.32, 150.00, 132.38, 132.28, 125.91, 120.34, 115.86, 115.65, 96.12, 77.23, 76.53, 74.46, 73.53, 63.44, 62.11, 59.59, 54.74, 41.51, 41.39, 38.65, 33.76, 32.62, 30.54, 30.07, 21.72, 19.95; ^{1}H-NMR ($CDCl_3$, 500 MHz), δ (ppm) 7.94 (2H, m, Ar-H), 7.06 (2H, m, Ar-H), 6.19 (1H, s, 17-CH_2), 6.11 (1H, s, 14-CH), 6.08 (1H, d, 6-OH), 5.51 (1H, s, 17-CH_2), 4.37, 4.09 (each 1H, d, J_A = J_B = 10.0 Hz, 20-CH_2), 3.90 (1H, s, 1-OH), 3.76 (1H, m, 6-CH), 3.52 (1H, m, 1-CH), 3.28 (1H, d, J = 9.5 Hz, 13-CH); MS (ESI) *m/z*: 487.2 $[M + H]^+$, 509.2 $[M + Na]^+$, 525.2 $[M + K]^+$.

Compound **8**: white solid, 61 % yield, m.p. 228-230 °C; IR (KBr) υ_{max} 3431, 3315, 2947, 1712, 1648, 1592, 1449, 1290, 1097, 1062, 969, 948, 911, 763, 733 cm^{-1}; ^{13}C-NMR ($CDCl_3$, 100 MHz), δ (ppm) 206.58, 164.26, 161.29, 149.96, 131.86, 130.16, 125.43, 120.56, 120.37, 116.54, 96.07, 77.23, 76.55, 74.58, 73.52, 63.45, 62.16, 59.52, 54.79, 41.53, 41.39, 38.66, 33.74, 32.63, 30.56, 30.05, 21.73, 19.96; ^{1}H-NMR ($CDCl_3$, 500 MHz), δ (ppm) 7.71 (1H, d, J = 10.0 Hz, Ar-H), 7.62 (1H, d, J = 10.0 Hz, Ar-H), 7.37 (1H, m, Ar-H), 7.23 (1H, m, Ar-H), 6.21 (1H, s, 17-CH_2), 6.12 (1H, s, 14-CH), 6.09 (1H, d, J = 10.0 Hz, 6-OH), 5.53 (1H, s, 17-CH_2), 4.37, 4.10 (each 1H, d, J_A = J_B = 10.0 Hz, 20-CH_2), 3.87 (1H, s, 1-OH), 3.75 (1H, m, 6-CH), 3.53 (1H, m, 1-CH), 3.28 (1H, d, J = 10.0 Hz, 13-CH); MS (ESI) *m/z*: 487.2 $[M + H]^+$, 509.2 $[M + Na]^+$, 525.2 $[M + K]^+$.

Compound **9**: white solid, 27 % yield, m.p. 242-244 °C; IR (KBr) υ_{max} 3446, 3350, 2960, 2926, 2852, 1724, 1712, 1649, 1257, 1163, 1132, 1088, 1060, 1025, 764, 677 cm^{-1}; ^{13}C-NMR ($CDCl_3$, 100 MHz), δ (ppm) 206.73, 164.28, 149.77, 135.10, 132.44, 124.26, 120.22, 117.68, 117.15, 96.11, 76.56, 74.79, 73.44, 63.45, 62.27, 59.13, 54.78, 41.52, 41.31, 38.60, 33.65, 32.67, 30.59, 29.96, 21.77, 19.98; ^{1}H-NMR ($CDCl_3$, 300 MHz), δ (ppm) 7.90 (1H, d, J = 7.6 Hz, Ar-H), 7.53 (1H, d, J = 7.2 Hz, Ar-H), 7.18 (1H, m, Ar-H), 7.10

(1H, m, Ar-H), 6.17 (1H, s, 17-CH_2), 6.12 (1H, s, 14-CH), 6.07 (1H, s, 6-OH), 5.49 (1H, s, 17-CH_2), 4.49 (1H, s, 1-OH), 4.36, 4.11 (each 1H, d, $J_A = J_B$ = 10.0 Hz, 20-CH_2), 3.78 (1H, m, 6-CH), 3.54 (1H, m, 1-CH), 3.68 (1H, d, J = 9.0 Hz, 13-CH); MS (ESI) *m*/*z*: 487.2 $[M + H]^+$.

Compound **10**: white solid, 28 % yield, m.p. 142-144 °C; IR (KBr) υ_{max} 3421, 2924, 2852, 2361, 1735, 1712, 1646, 1629, 1382, 1222, 1061, 1046, 969, 912, 776 cm^{-1}; ^{13}C-NMR ($CDCl_3$, 100 MHz), δ (ppm) 206.23, 160.77, 149.77, 120.55, 114.81, 113.52, 95.85, 76.55, 74.77, 73.48, 63.45, 62.29, 59.17, 54.85, 41.57, 41.34, 38.62, 33.70, 32.65, 30.59, 29.98, 21.76, 20.01; ^1H-NMR ($CDCl_3$, 300 MHz), δ (ppm) 7.60 (1H, m, Ar-H), 6.20 (1H, s, 17-CH_2), 6.17 (1H, s, 14-CH), 6.09 (1H, d, J = 10.8 Hz, 6-OH), 5.55 (1H, s, 17-CH_2), 4.37, 4.10 (each 1H, d, $J_A = J_B$ = 10.2 Hz, 20-CH_2), 3.80 (1H, s, 1-OH), 3.73 (1H, m, 6-CH), 3.53 (1H, m, 1-CH), 3.26 (1H, d, J = 10.2 Hz, 13-CH); MS (ESI) *m*/*z*: 541.2 $[M + H]^+$.

Compound **11**: white solid, 57 % yield, m.p. 114-116 °C; IR (KBr) υ_{max} 3448, 2922, 2851, 1735, 1711, 1641, 1458, 1384, 1274, 1168, 1061, 785, 765, 750 cm^{-1}; ^{13}C-NMR ($CDCl_3$, 100 MHz), δ (ppm) 206.17, 169.91, 149.27, 133.82, 131.85, 129.49, 128.81, 128.42, 128.12, 126.43, 125.83, 125.65, 123.48, 119.67, 96.14, 77.23, 73.83, 73.44, 63.35, 61.72, 59.77, 54.39, 41.24, 40.92, 39.62, 38.60, 33.74, 32.49, 30.14, 30.08, 21.62, 19.71; ^1H-NMR ($CDCl_3$, 300 MHz), δ (ppm) 7.77 (3H, m, Ar-H), 7.48 (1H, s, Ar-H), 7.38 (3H, m, Ar-H), 6.00 (1H, d, J = 9.9 Hz, 6-OH), 5.88 (1H, s, 17-CH_2), 5.79 (1H, s, 14-CH), 5.03 (1H, s, 17-CH_2), 4.26, 4.04 (each 1H, d, $J_A = J_B$ = 9.9 Hz, 20-CH_2), 3.98 (1H, s, 1-OH), 3.72 (1H, m, 6-CH), 3.45 (1H, m, 1-CH), 3.01 (1H, d, J = 9.5 Hz, 13-CH); MS (ESI) *m*/*z*: 533.3 $[M + H]^+$, 550.3 $[M + NH_4]^+$.

Compound **12**: yellow solid, 47 % yield, m.p. 250-252 °C; IR (KBr) υ_{max} 3547, 3413, 3239, 2926, 1712, 1647, 1529, 1400, 1306, 1242, 1190, 1145, 1078, 1061, 752 cm^{-1}; δ (ppm) 206.67, 164.35, 147.23, 137.04, 127.38, 126.49, 125.83, 122.72, 120.96, 120.53, 111.97, 109.62, 96.27, 77.22, 76.29, 74.53, 73.53, 63.53, 62.12, 59.70, 54.67, 41.60, 41.39, 38.63, 33.78, 32.58, 30.48, 30.10, 21.70, 19.89; ^1H-NMR ($CDCl_3$, 500 MHz), δ (ppm) 9.11 (1H, s, NH), 7.44 (5H, m, Ar-H), 6.20 (1H, s, 17-CH_2), 6.06 (1H, s, 6-OH), 5.53 (1H, s, 14-CH), 5.30 (1H, s, 17-CH_2), 4.40, 4.11 (each 1H, d, $J_A = J_B$ = 9.5 Hz, 20-CH_2), 3.83 (1H, s, 1-OH), 3.53 (1H, m, 6-CH), 3.42 (1H, m, 1-CH), 3.38 (1H, d, J = 9.6 Hz, 13-CH); MS (ESI) *m*/*z*: 508.2 $[M + H]^+$, 530.5 $[M + Na]^+$, 506.2 $[M - H]^-$.

Compound **14**: white solid, 44 % yield, m.p. 104-106 °C; IR (KBr) υ_{max} 3400, 2955, 2923, 2851, 1731, 1461, 1384, 1275, 1079, 767, 760, 743 cm^{-1}; ^{13}C-NMR ($CDCl_3$, 100 MHz), δ (ppm) 211.78, 204.91, 174.88, 149.37, 121.94, 97.10, 75.46, 72.95, 64.94, 61.10, 60.37, 50.75, 48.54, 43.39, 38.55, 35.86, 30.53, 29.93, 29.70, 29.37, 25.84, 25.71, 23.30, 19.01; ^1H-NMR ($CDCl_3$, 300 MHz), δ (ppm) 6.13 (1H, s, 17-CH_2), 6.09 (1H, s, 6-OH), 5.78 (1H, s, 14-CH), 5.47 (1H, s, 17-CH_2), 4.44 (1H, s, 7-OH), 4.30, 4.08 (each 1H, d, J_A = J_B = 10.5 Hz, 20-CH_2), 3.79 (1H, m, 6-CH), 3.19 (1H, d, J = 9.9 Hz, 13-CH); MS (ESI) *m*/*z*: 459.2 $[M + H]^+$.

Compound **15**: yellow oil, 40 % yield; IR (KBr) υ_{max} 3512, 3393, 2954, 2924, 2851, 1735, 1715, 1695, 1245, 1160, 1130, 1086, 1057, 910, 804 cm^{-1}; ^3C-NMR ($CDCl_3$, 100 MHz), δ (ppm) 211.77, 204.85, 174.22, 149.34, 121.93, 97.10, 75.27, 72.95, 64.92, 61.08, 60.32, 50.72, 48.53, 43.19, 41.27, 38.53, 35.84, 32.88, 30.54, 29.91, 29.59, 28.81, 25.52, 25.26, 25.14, 23.30, 19.02; ^1H-NMR ($CDCl_3$, 300 MHz), δ (ppm) 6.24 (1H, s, 17-CH_2), 5.78 (1H, s, 14-CH), 5.59 (1H, s, 17-CH_2), 4.41 (1H, s, 7-OH), 4.29, 4.04 (each 1H, d, J_A = J_B = 15.5 Hz, 20-CH_2), 3.82 (1H, m, 6-CH), 3.13 (1H, d, J = 15.5 Hz, 13-CH); MS (ESI) *m*/*z*: 473.3 $[M + H]^+$, 495.3 $[M + Na]^+$.

Compound **16**: yellow oil, 36 % yield; IR (KBr) υ_{max} 3390, 2955, 2925, 2855, 1709, 1644, 1463, 1395, 1377, 1212, 1163, 1080, 1057, 805, 736 cm^{-1}; ^{13}C-NMR ($CDCl_3$, 100 MHz), δ (ppm) 211.72, 204.94, 172.15, 149.38, 122.03, 97.02, 75.08, 73.04, 64.93, 61.14, 60.19, 50.73, 48.53, 41.33, 38.51, 35.83, 34.54, 32.89, 31.61, 30.51, 29.70, 28.94, 28.83, 24.60, 23.27, 22.57, 19.04, 14.06; ^1H-NMR ($CDCl_3$, 300 MHz), δ (ppm) 6.21 (1H, s, 17-CH_2), 5.84 (1H, s, 14-CH), 5.61 (1H, s, 17-CH_2), 4.43 (1H, s, 7-OH), 4.29, 4.04 (each 1H, d, $J_A = J_B$ = 10.5 Hz, 20-CH_2), 3.82 (1H, m, 6-CH), 3.14 (1H, d, J = 9.0 Hz, 13-CH); MS (ESI) *m*/*z*: 489.2 $[M + H]^+$, 506.2 $[M + NH_4]^+$.

Compound **17**: white solid, 47 % yield, m.p. 95-97 °C; IR (KBr) υ_{max} 3531, 3390, 2909, 2852, 1708, 1644, 1268, 1234, 1214, 1182, 1078, 1057, 975, 941 cm^{-1}; ^{13}C-NMR ($CDCl_3$, 100 MHz), δ (ppm) 211.80, 204.78, 172.81, 149.34, 121.77, 97.16, 75.66, 72.89, 64.96, 61.05, 60.56, 50.80, 48.57, 41.18, 40.84, 38.62, 38.38,

36.41, 36.18, 35.90, 32.91, 31.93, 29.87, 29.70, 29.37, 27.82, 27.65, 23.34, 22.70, 18.99; ^{1}H-NMR ($CDCl_3$, 300 MHz), δ (ppm) 6.25 (1H, s, 17-CH_2), 5.74 (1H, s, 14-CH), 5.60 (1H, s, 17-CH_2), 5.46 (1H, d, J = 11.7 Hz, 6-OH), 4.56 (1H, s, 7-OH), 4.29, 4.05 (each 1H, d, J_A = J_B = 10.0 Hz, 20-CH_2), 3.84 (1H, m, 6-CH), 3.12 (1H, d, J = 9.3 Hz, 13-CH); MS (ESI) *m*/*z*: 525.2 [M + H]$^+$.

Compound **18**: white solid, 18 % yield, m.p. 88-90 °C; IR (KBr) υ_{max} 3398, 2959, 2874, 1709, 1644, 1465, 1282, 1244, 1167, 1128, 1079, 1066, 1017, 966 cm^{-1}; ^{13}C-NMR ($CDCl_3$, 100 MHz), δ (ppm) 211.51, 205.02, 164.25, 149.52, 133.27, 130.22, 125.39, 122.29, 96.86, 74.73, 73.53, 64.94, 61.59, 59.59, 50.72, 48.53, 41.69, 38.28, 35.69, 32.81, 30.46, 30.08, 23.11, 19.27; ^{1}H-NMR ($CDCl_3$, 300 MHz), δ (ppm) 8.04 (2H, d, J = 8.4 Hz, Ar-H), 7.65 (2H, d, J = 8.1 Hz, Ar-H), 6.31 (1H, s, 17-CH_2), 6.18 (1H, s, 14-CH), 5.66 (1H, s, 17-CH_2), 5.38 (1H, d, J = 11.7 Hz, 6-OH), 4.35, 4.04 (each 1H, d, J_A = J_B = 10.4 Hz, 20-CH_2), 3.75 (1H, s, 7-OH), 3.74 (1H, m, 6-CH), 3.23 (1H, d, J = 9.6 Hz, 13-CH); MS (ESI) *m*/*z*: 535.1 [M + H]$^+$, 552.1 [M + NH_4]$^+$.

Compound **20**: white solid, 19 % yield, m.p. 124-126 °C; IR (KBr) υ_{max} 3398, 2924, 2853, 1709, 1643, 1592, 1287, 1270, 1154, 1097, 1079, 1001, 909 cm^{-1}; ^{13}C-NMR ($CDCl_3$, 100 MHz), δ (ppm) 211.62, 205.10, 164.28, 161.21, 149.50, 132.05, 130.01, 125.56, 122.29, 120.34, 116.55, 96.87, 74.82, 73.41, 64.91, 61.54, 59.68, 50.73, 48.54, 41.66, 38.33, 35.73, 32.83, 30.46, 30.06, 23.15, 19.23; ^{1}H-NMR ($CDCl_3$, 300 MHz), δ (ppm) 7.70 (1H, m, Ar-H), 7.61 (1H, d, J = 8.7 Hz, Ar-H), 7.37 (1H, m, Ar-H), 7.23 (1H, m, Ar-H), 6.31 (1H, s, 17-CH_2), 6.14 (1H, s, 14-CH), 5.65 (1H, s, 17-CH_2), 5.40 (1H, d, J = 11.7 Hz, 6-OH), 4.35, 4.04 (each 1H, d, J_A = J_B = 10.0 Hz, 20-CH_2), 3.87 (1H, s, 7-OH), 3.77 (1H, m, 6-CH), 3.24 (1H, d, J = 9.9 Hz, 13-CH); MS (ESI) *m*/*z*: 485.2 [M + H]$^+$.

Compound **21**: white solid, 38 % yield, m.p. 108-110 °C; IR (KBr) υ_{max} 3408, 2957, 2929, 2873, 1711, 1643, 1613, 1244, 1159, 1132, 1082, 1051, 910 cm^{-1}; ^{13}C-NMR ($CDCl_3$, 100 MHz), δ (ppm) 211.75, 204.78, 162.85, 160.58, 149.26, 135.12, 132.45, 124.24, 122.15, 117.81, 117.17, 96.91, 76.11, 73.20, 64.97, 61.45, 60.22, 50.85, 48.60, 41.38, 38.54, 35.86, 32.90, 30.53, 30.06, 23.28, 19.13; ^{1}H-NMR ($CDCl_3$, 300 MHz), δ (ppm) 7.88 (1H, m, Ar-H), 7.52 (1H, m, Ar-H), 7.19 (1H, t, J = 7.5 Hz, Ar-H), 7.09 (1H, t, J = 7.5 Hz, Ar-H), 6.28 (1H, s, 17-CH_2), 6.08 (1H, s, 14-CH), 5.63 (1H, s, 17-CH_2), 5.53 (1H, d, J = 11.1 Hz, 6-OH), 4.37 (1H, s, 7-OH), 4.35, 4.07 (each 1H, d, J_A = J_B = 10.5 Hz, 20-CH_2), 4.05 (1H, m, 6-CH), 3.32 (1H, d, J = 9.0 Hz, 13-CH); MS (ESI) *m*/*z*: 485.2 [M + H]$^+$.

Compound **22**: white solid, 42 % yield, m.p. 199-201 °C; IR (KBr) υ_{max} 3406, 2959, 2875, 1707, 1643, 1629, 1527, 1286, 1212, 1080, 1051, 1036, 968, 910 cm^{-1}; ^{13}C-NMR ($CDCl_3$, 100 MHz), δ (ppm) 211.36, 204.59, 149.30, 122.38, 131.48, 96.75, 75.09, 73.56, 65.02, 61.63, 59.35, 50.76, 48.52, 41.66, 38.24, 35.69, 32.84, 30.47, 30.07, 23.07, 19.28; ^{1}H-NMR ($CDCl_3$, 300 MHz), δ (ppm) 7.56 (1H, m, Ar-H), 6.31 (1H, s, 17-CH_2), 6.21 (1H, s, 14-CH), 5.68 (1H, s, 17-CH_2), 5.39 (1H, d, J = 11.7 Hz, 6-OH), 4.35, 4.04 (each 1H, d, J_A = J_B = 10.5 Hz, 20-CH_2), 3.92 (1H, s, 7-OH), 3.77 (1H, m, 6-CH), 3.21 (1H, d, J = 9.0 Hz, 13-CH); MS (ESI) *m*/*z*: 539.1 [M + H]$^+$.

Compound **23**: white solid, 41 % yield, m.p. 192-194 °C; IR (KBr) υ_{max} 3402, 2953, 2923, 1853, 1702, 1639, 1269, 1200, 1178, 1161, 1132, 1085, 1052, 976, 951, 911 cm^{-1}; ^{13}C-NMR ($CDCl_3$, 100 MHz), δ (ppm) 211.64, 204.56, 169.86, 148.97, 133.79, 131.93, 129.70, 128.70, 128.27, 128.12, 126.36, 125.77, 125.51, 123.67, 121.73, 96.87, 75.33, 73.09, 64.86, 61.18, 59.94, 50.59, 48.49, 41.18, 39.42, 38.45, 35.77, 32.84, 30.49, 29.72, 23.22, 19.04; ^{1}H-NMR ($CDCl_3$, 500 MHz), δ (ppm) 7.55 (7H, m, Ar-H), 6.00 (1H, s, 17-CH_2), 5.82 (1H, s, 14-CH), 5.47 (1H, d, J = 11.4 Hz, 6-OH), 5.29 (1H, s, 17-CH_2), 4.24, 4.11 (each 1H, d, J_A = J_B = 10.4 Hz, 20-CH_2), 3.80 (1H, s, 7-OH), 3.77 (2H, s, 12-CH_2), 3.76 (1H, m, 6-CH), 2.96 (1H, d, J = 8.7 Hz, 13-CH); MS (ESI) *m*/*z*: 531.1 [M + H]$^+$, 548.2 [M + NH_4]$^+$.

Compound **24**: colorless oil, 41 % yield; IR (KBr) υ_{max} 3398, 2956, 2926, 2871, 1704, 1642, 1241, 1190, 1147, 1080, 1050, 910 cm^{-1}; ^{13}C-NMR ($CDCl_3$, 100 MHz), δ (ppm) 211.64, 205.11, 160.31, 149.32, 137.06, 127.32, 126.54, 125.78, 122.68, 122.44, 120.94, 112.00, 109.68, 97.10, 75.41, 73.48, 65.03, 61.51, 60.09, 50.84, 48.55, 41.49, 38.42, 35.79, 32.88, 30.51, 30.03, 23.22, 19.16; ^{1}H-NMR ($CDCl_3$, 300 MHz), δ (ppm) 9.09 (1H, s, -NH), 7.62 (1H, d, J = 7.0 Hz, Ar-H), 7.40 (1H, d, J = 5.3 Hz, Ar-H), 7.20 (3H, m, Ar-H), 6.30 (1H, s, 17-CH_2), 6.06 (1H, s, 14-CH), 5.64 (1H, s, 17-CH_2), 5.52 (1H, d, J = 11.7 Hz, 6-OH), 4.35, 4.05

(each 1H, d, $J_A = J_B = 11.5$ Hz, 20-CH_2), 4.33 (1H, s, 7-OH), 3.82 (1H, m, 6-CH), 3.33 (1H, m, 13-CH); MS (ESI) *m*/*z*: 508.2 $[M + H]^+$.
